# White matter disruptions related to inattention and autism spectrum symptoms in tuberous sclerosis complex

**DOI:** 10.1016/j.nicl.2022.103163

**Published:** 2022-08-25

**Authors:** Lucy D. Vanes, Charlotte Tye, Jacques-Donald Tournier, Anna J.E. Combes, Elizabeth Shephard, Holan Liang, Gareth J. Barker, Chiara Nosarti, The TS2000 Study Team, Patrick Bolton

**Affiliations:** a Department of Neuroimaging, Institute of Psychiatry, Psychology, & Neuroscience, King’s College London, UK; b Department of Perinatal Imaging and Health, School of Biomedical Engineering & Imaging Sciences, King’s College London, UK; c Department of Psychology, Institute of Psychiatry, Psychology, & Neuroscience, King’s College London, UK; d Department of Biomedical Engineering, School of Biomedical Engineering & Imaging Sciences, King’s College London, UK; e Department of Child & Adolescent Psychiatry, Institute of Psychiatry, Psychology, & Neuroscience, King’s College London, UK; f Department of Psychiatry, Faculdade de Medicina, Universidade de São Paulo, Brazil

**Keywords:** ADHD, attention-deficit/hyperactivity disorder, ASD, autism spectrum disorder, CSD, constrained spherical deconvolution, E-Chess, Early Childhood Epilepsy Severity Scale, FBA, fixel-based analysis, FC, fibre cross-section, FD, fibre density, FDC, fibre density cross-section, FOD, fibre orientation distribution, FSIQ, full-scale IQ, ILF, inferior longitudinal fasciculus, FWE, family-wise error, SIFT, spherical-deconvolution informed filtering of tractograms, SLF, superior longitudinal fasciculus, SCI, Social Communication Index, SRS, Social Responsiveness Scale, RRB, Restricted, Repetitive Behaviours, TSC, Tuberous Sclerosis Complex, WASI, Wechsler Abbreviated Scale of Intelligence, Tuberous sclerosis complex, Fixel-based analysis, White matter, Autism, Inattention, Neurodevelopment

## Abstract

Tuberous sclerosis complex is a rare genetic multisystem condition that is associated with a high prevalence of neurodevelopmental disorders such as autism and attention-deficit/hyperactivity disorder. The underlying neural mechanisms of the emergence of these symptom domains in tuberous sclerosis complex remain unclear.

Here, we use fixel-based analysis of diffusion-weighted imaging, which allows for the differentiation between multiple fibre populations within a voxel, to compare white matter properties in 16 participants with tuberous sclerosis complex (aged 11–19) and 12 age and sex matched control participants. We further tested associations between white matter alterations and autism and inattention symptoms as well as cognitive ability in participants with tuberous sclerosis complex.

Compared to controls, participants with tuberous sclerosis complex showed reduced fibre density cross-section (FDC) in the dorsal branch of right superior longitudinal fasciculus and bilateral inferior longitudinal fasciculus, reduced fibre density (FD) in bilateral tapetum, and reduced fibre cross-section (FC) in the ventral branch of right superior longitudinal fasciculus. In participants with tuberous sclerosis complex, the extent of FDC reductions in right superior longitudinal fasciculus was significantly associated with autism traits (social communication difficulties and restricted, repetitive behaviours), whereas FDC reductions in right inferior longitudinal fasciculus were associated with inattention. The observed white matter alterations were unrelated to cognitive ability.

Our findings shed light on the fibre-specific biophysical properties of white matter alterations in tuberous sclerosis complex and suggest that these regional changes are selectively associated with the severity of neurodevelopmental symptoms.

## Highlights

• Density and cross-section of specific white matter fibres is reduced in TSC.
• Right SLF-I is implicated in autism spectrum symptoms in TSC.
• Bilateral ILF is implicated in ADHD symptoms in TSC.
• Fixel-based analysis is a useful tool to evaluate white matter changes in TSC.
• White matter changes may underlie neurodevelopmental disorder in TSC.

## 1 Introduction

Tuberous Sclerosis Complex (TSC) is a rare autosomal dominant condition characterised by benign tumorlike malformations (hamartomas) affecting several organ systems, including the brain, and concomitant neuropsychological manifestations such as seizures, developmental deficits, and neuropsychiatric disorders (Curatolo and Bombardieri, 2007, Curatolo et al., 2015, Muzykewicz et al., 2007). At the neural level, a hallmark of TSC is the presence of cortical tubers; regions of disorganised neural tissue with prominent oversized cells (Orlova and Crino, 2010). While cortical tubers are thought to contribute to the emergence of neural dysfunction (such as epilepsy) in TSC (Kassiri et al., 2011), tuber load or location alone are insufficient predictors of the diverse neurological, cognitive, and psychological outcomes of the condition (Jansen et al., 2008, Wong, 2006).

Rates of developmental and psychiatric disorders are markedly elevated in the TSC population (Curatolo et al., 2015, Muzykewicz et al., 2007). In particular, the prevalence of attention-deficit/hyperactivity disorder (ADHD) and autism spectrum disorder (ASD) in TSC have been variably estimated to be as high as 60 % (Muzykewicz et al., 2007, de Vries et al., 2007, Hunt, 1993), with co-occurring ADHD and ASD also commonly observed (Gillberg et al., 1994, Gupta et al., 2020). However, the underlying cause of the emergence of these neurodevelopmental conditions in TSC is still unclear. Disruptions of structural connectivity in key circuits due to the presence of cortical tubers has been suggested (Tsai et al., 2021), with TSC participants showing pervasive abnormalities in white matter microstructure (Taoka et al., 2020, Peters et al., 2012) and global organisation (Tsai et al., 2021) compared to control participants without TSC, and tuber load itself correlating with indices of white matter pathology such as reduced fractional anisotropy (Simao et al., 2010) and increased mean diffusivity (Im et al., 2016). Indeed, larger white matter changes have been observed in TSC participants with ASD compared to TSC participants without ASD (Peters et al., 2012), suggesting a direct relationship between abnormal white matter tract integrity and the emergence of ASD symptomatology. Similar observations have been made on a functional level, with early markers of altered functional connectivity being most pronounced in infants with TSC who go on to develop ASD (Dickinson et al., 2019), and reports that changes in functional network topology in co-occurring TSC and ASD are comparable to those seen in idiopathic ASD (Peters et al., 2013). While studies investigating the neural underpinning of ADHD in TSC are comparably scarce, previous work from our group recently showed that functional hypo-connection of neural networks in TSC is associated both with symptoms of ASD and ADHD (Shephard et al., 2022). However, there remains a need for investigations into the corresponding structural correlates of both of these symptom domains in TSC, in particular focusing on properties of white matter fibres which underpin the structural architecture of the brain.

Diffusion imaging has been successfully applied in TSC to assess white matter microstructure, with findings converging on dysconnectivity due to structurally compromised and disorganised axons (Im et al., 2016, Taoka et al., 2020, Peters et al., 2012). In particular, TSC participants display reduced fractional anisotropy and increased mean diffusivity in numerous white matter tracts (Peters et al., 2012, Im et al., 2016, Krishnan et al., 2010), consistent with a reduced capacity for efficient information transfer between disparate regions. These metrics appear to be particularly altered in regions with cortical tubers and other lesions (Tsai et al., 2021). Interestingly, recent work has demonstrated the utility of advanced diffusion models in identifying reduced neurite density in TSC across widespread regions, including deep white matter tracts such as the superior longitudinal fasciculus (Taoka et al., 2020). Graph theoretical analyses based on diffusion imaging suggest that compromised white matter integrity results in increased segregation of neural networks within hemispheres (Im et al., 2016) and a concomitant reduction of global efficiency (Tsai et al., 2021). However, a disadvantage of conventional diffusion imaging analysis procedures is the inability to characterise microstructural properties in regions of multiple fibre orientations (“crossing” fibres), which hampers the interpretability of the derived diffusion indices. Furthermore, these standard indices do not allow for an assessment of the underlying biophysical properties of individual fibres, such as fibre diameter or density. In order to address these shortcomings, fixel-based analysis (FBA) has been proposed as a novel analytic framework (Raffelt et al., 2017) which allows multiple fibre populations within a single voxel to be resolved. FBA can therefore be used to characterise micro- and macrostructural properties of individual fibre populations within a voxel (i.e., fixels); in particular, FBA yields fixel-specific measures of fibre density (FD), fibre cross-section (FC), and a combined measure of density and cross-section (FDC). To date, measures of fibre density and cross-section have not been investigated in TSC, but may shed light on the biophysical processes underlying neural dysfunction and behavioural manifestations thereof.

Despite evidence suggesting that a diagnosis of neurodevelopmental disorders in TSC is associated with more severe white matter abnormalities (Tsai et al., 2021), studies thus far have not investigated the association between white matter abnormalities and ADHD or ASD symptomatology in TSC dimensionally. It is therefore unclear whether the extent of white matter disruption in TSC is related to the severity of these types of psychopathology, rendering individual prediction of psychiatric outcomes difficult. Both ASD and ADHD symptomatology can occur at subthreshold levels without necessitating a formal diagnosis, but can nevertheless cause significant difficulties in daily life (Christ et al., 2010, Hong et al., 2014). Importantly, taking into account expression of these symptoms across a dimensional spectrum can shed light on the underlying aetiology of more severe forms of the conditions. In particular, as individual outcomes in the TSC population can differ substantially, it is of increasing importance to identify potential biomarkers that may be indicative of the severity of neurodevelopmental outcomes in these patients.

The aim of the current study therefore was to evaluate white matter properties in a sample of TSC participants using FBA, allowing for an advanced assessment of micro- and macroscopic changes of connective white matter fibres throughout the whole brain. Given previous findings of disrupted white matter connectivity in TSC, we expected to observe reduced fibre density or cross-section (or both) in individual white matter tracts in TSC participants compared to controls; however, given that – to our knowledge – FBA has not been previously employed to study white matter in TSC, we conducted our analyses on an exploratory whole-brain level in order to detect the most affected regions using this technique. Having identified regions in which TSC participants show compromised white matter architecture compared to control participants without TSC, we next aimed to test whether the extent of the disruption was related to the expression of ADHD (inattention / hyperactivity) or ASD (social communication / restricted repetitive behaviours) symptomatology. We expected participants with TSC to show significantly elevated symptoms of ADHD and ASD compared to controls, and that select white matter alterations would be associated with these behavioural difficulties within the TSC group. Given the implication of cognitive deficits in neurodevelopmental disorders and TSC, we also assessed associations between white matter disruption and cognitive ability in these patients in order to test explicitly whether observed associations were specific to psychopathology, or rather reflective of general cognitive difficulties. Finally, we explored whether any of the observed white matter abnormalities were significantly associated with key clinical variables such as tuber load or history and severity of epilepsy.

## 2 Materials and methods

### 2.1 Sample

Participants were 16 individuals (aged 11–19) with TSC enrolled in the Tuberous Sclerosis 2000 (TS 2000) study, a prospective longitudinal study of 125 children in the United Kingdom diagnosed with TSC between 2001 and 2005. Children were originally recruited aged 0–16 via the UK Tuberous Sclerosis Association and UK-based paediatricians, paediatric neurologists, and clinical geneticists. Phase 1 of the study (2001–2005) (Yates et al., 2011) included medical, genotypic, and intellectual assessments, and further developmental and intellectual assessments were undertaken in Phase 2 (2013–2015) (Tye et al., 2020). A total of 48 participants participated in the current follow up (Phase 3; 2015–2017), involving further psychiatric and cognitive assessments as well as administration of self- and parent-rated questionnaires. A subsample of 19 individuals underwent magnetic resonance imaging (MRI), of whom 16 with complete diffusion imaging data were included in the analysis.

In addition, 23 typically developing young people (aged 10–25) without a diagnosis of TSC or symptoms of ASD, ADHD, intellectual disability, or epilepsy, were recruited as a control group via local advertisements and word-of-mouth. MRI scans were available for 12 of these control participants.

Ethical approval was provided by the NHS National Research Ethics Service (NHS Edgbaston REC 00/7/061) and study procedures were conducted in line with the Declaration of Helsinki (World Medical Association, as amended 2013). All participants or their parent/legal guardian (for those aged < 16) provided written informed consent. All participants also provided verbal assent to participate in each study measure.

### 2.2 Assessments

Participants underwent cognitive assessment using the two-subtest version of the Wechsler Abbreviated Scale of Intelligence Second Edition (WASI-II) (Wechsler, 2011). Age- and sex-normed full-scale intelligence quotient (FSIQ) was used in further analyses. Autism symptoms were assessed using the parent-rated Social Responsiveness Scale - Second Edition (SRS-2) (Constantino and Gruber, 2012), from which the Social Communication Index (SCI) and Restricted, Repetitive Behaviours (RRB) subscales were derived. ADHD symptoms were assessed using the parent-rated Conners 3 (Conners, 2008), from which the Inattention and Hyperactivity/Impulsivity subscales were derived. Information on seizures, epilepsy severity, and medication status was collected for three months prior to study participation via parental interview (Yates et al., 2011) and a seizure diary, which was subsequently scored using the Early Childhood Epilepsy Severity Scale (E-Chess) (Humphrey et al., 2008). Higher scores on these measures reflect higher cognitive ability and severity of autism, ADHD and epilepsy symptoms.

### 2.3 MRI acquisition

Participants underwent MRI on one of two nominally identical 3 T GE MR750 scanners with an 8-channel head coil (GE Healthcare, USA), situated at King’s College London or at the NIHR-Wellcome Trust King’s Clinical Research Facility, King’s College Hospital. Multi-shell diffusion-weighted images were acquired using a spin-echo echo planar imaging sequence using identical protocols with the following parameters: TR/TE = 4000 ms/79.80 ms, voxel size = 2 mm isotropic, flip angle = 90°, matrix size = 128 × 128, field of view = 256 × 256 mm^2^. Diffusion-weighted gradients were applied along 195 non-collinear directions using b-values of 0 (13), 500 (30), 1500 (60), and 3000 (92) s/mm^2^. A reverse phase encoded b0 volume was also acquired to correct for susceptibility-induced distortions. All images were visually inspected for head motion artefacts evidenced by slice-dropout, signal loss or excessive venetian blinding artefacts. Images were inspected on all three planes and no participants were excluded due to excessive volume corruption. Output images from pre-processing were visually inspected for further artefacts and all available subjects were retained.

### 2.4 Image pre-processing and fixel-based analysis

Diffusion images were processed using MRTrix3 (Tournier et al., 2019). Data were denoised using *dwidenoise*, and motion and susceptibility distortion correction applied using FSL’s *topup* (Andersson et al., 2003) and *eddy*; (Andersson and Sotiropoulos, 2016) as implemented in MRTrix3′s *dwifslpreproc*. Additional bias field correction was applied using *dwibiascorrect*. Images were subsequently upsampled to 1.2 mm isotropic voxel resolution. After preprocessing, multi-shell multi-tissue constrained spherical deconvolution (CSD) was applied to estimate the fibre orientation distribution (FOD) within each voxel using the *msmt_csd* algorithm (Jeurissen et al., 2014). Global intensity normalisation was applied in order to correct for intensity inhomogeneities (Raffelt et al., 2017). A study-specific white matter FOD template was generated from all TSC and control participants using symmetric diffeomorphic registration (Raffelt et al., 2011) and each participant’s FOD image was individually registered to the template. In order to rule out systematic bias towards one group within the template, we derived the Jacobian deformations of the individual registrations to the template and compared these between groups using FSL’s *randomise* (5000 permutations). No significant group differences were observed (two-sided *t*-test *p* >.05), suggesting the template was unbiased by group. FOD images were segmented to estimate fixels and their apparent fibre density at the participant and template level, and participant-level fixels were reoriented to the corresponding fixels in template space (Smith et al., 2013). Whole-brain tractography was performed on the FOD template, generating 20 million tracts, which were subsequently filtered down to 2 million tracts using spherical-deconvolution informed filtering of tractograms (SIFT) (Smith et al., 2013).

The following fixel-based metrics were derived: FD, a microstructural measure of fibre density of specific fibre bundles within a voxel, which is derived as the integral of the FOD; FC, a macrostructural measure of the cross-section of specific fibre bundles on a logarithmic scale, which is derived from the Jacobian determinant of the non-linear registration field from the individual FOD to the template FOD, whereby the natural logarithm (log-FC) is used for further analyses (in order to ensure that data is centred around zero and normally distributed); and FDC, a combined measure of fibre density and cross-section that is thought to be more sensitive to a fibre bundle’s ability to relay information (Raffelt et al., 2017).

### 2.5 Tuber load

In TS participants, tuber load was ascertained via inspection of clinical MRI scans from Phase 1 (or, where available, Phases 2 and 3) of the study by three consultant neuroradiologists. The scans were reviewed and rated without knowledge of other clinical details using a pre-specified coding system recording the number and lobar location of cortical tubers (see Supplementary Materials for further details). The inter-rater reliability of this procedure has previously been shown to be acceptable (Bolton et al., 2002). Tuber count was summed across the whole brain as a measure of total tuber load. Anatomical scans were reviewed by a consultant radiologist to rule out any clinically relevant abnormalities in the control group.

### 2.6 Statistical analysis

The analysis was conducted in three stages. First, we identified behavioural variables of interest, defined as those on which TSC participants differed significantly from controls. Next, we identified white matter regions showing significant differences in FBA metrics between TSC and control groups. Finally, we tested – in the TSC group only – whether white matter properties within these regions were significantly associated with the behavioural difficulties observed. Importantly, by testing brain-behaviour associations only within the TSC group, these associations remained statistically independent (i.e., unbiased) with respect to group differences observed in the behaviours and the brain regions of interest ascertained in the earlier stages.

***Behavioural group differences.*** We tested for group differences in FSIQ, SRS-SCI, SRS-RRB, Conners Inattention, and Conners Hyperactivity/Impulsivity, respectively, using multiple regression analyses controlling for age and sex.

***Neural group differences.*** Whole-brain FBA metrics (FDC, FD, and log-FC) were compared between groups using non-parametric permutation testing (5000 permutations) with connectivity-based fixel enhancement (Raffelt et al., 2015) to derive family-wise error (FWE) corrected fixel-wise *p*-values. Age, sex, and scanner were included as covariates of no interest. Where significant group differences were found in FDC, FD, or log-FC, mean values of the relevant metric, as well as mean beta and *p*-values for the group effect, were extracted for each subject from the significant region. Where a group effect showed significant fixels in several anatomically separate white matter tracts, mean values were extracted for each tract-specific region separately. This was achieved by manually drawing a region of interest around the significant fixels within an individual tract (using the MRView ROI tool) and intersecting it with the thresholded FWE-corrected *p*-value map in order to obtain separate masks for each tract-specific effect. A significance threshold of *p* =.05 was used throughout. FDC is likely to be particularly sensitive to detecting group differences, but effects of FDC may nevertheless be driven predominantly by either fibre microstructure (FD) or macrostructure (FC); therefore, mean FD and log-FC were also extracted from regions showing significant differences in FDC in order to explore which of these properties most influenced the observed group differences in FDC.

***Brain-behaviour associations in TSC participants***. Separate linear models were run (within the TSC group only) to test whether any of the extracted mean FBA metrics were significantly associated with the identified behavioural variables of interest, controlling for effects of age, sex, and scanner. Effects were evaluated against a Bonferroni-corrected significance threshold of *p* =.05/4 = 0.0125 (accounting for four identified behavioural variables of interest: FSIQ, SRS-SCI, SRS-RRB, and Conners Inattention). We did not undertake further correction for number of regions tested, but uncorrected p-values are reported throughout to allow for an evaluation against alternative significance thresholds. For completeness, we also tested brain-behaviour relationships in the control group, though these were of secondary interest.

Finally, we tested for associations between fixel-based metrics and clinical variables including tuber load, history and severity of epilepsy, and age of seizure onset.

### 2.7 Sensitivity analyses

To ensure that the observed effects were not a result of confounding effects of total intracranial volume (TIV), in-scanner head motion, or medication status, we conducted additional analyses controlling for these variables. Extracted fixel-based metrics were tested for group differences controlling for age, sex, and scanner (as in the whole-brain analysis) with the addition of TIV or head motion as additional covariate of non-interest. Group differences (in behavioural and fixel-based metrics) were also tested after excluding 4 TSC participants currently prescribed medication. Detailed results of these analyses can be found in Supplementary Materials. Brain-behaviour analyses in the TSC group were also re-run with the addition of TIV, head motion, or medication status as additional covariate of non-interest. Results of these analyses can be found in Supplementary Table S2. In brief, inclusion of TIV, in-scanner motion, or medication status did not alter any of the reported results.

## 3 Results

Sample characteristics can be found in Table 1. Groups did not differ significantly in terms of age, *t*(14.5) = 0.26, *p* =.793, sex distribution, *χ*^2^(1) = 0.62, *p* =.433, TIV, *t*(26) = −0.25, *p* =.805, or head motion, *t*(26) = −0.18, *p* =.857.

**Table 1:** Demographic and clinical characteristics of the Tuberous Sclerosis (TSC) and Control groups.

	TSC (N = 16)		Control (N = 12)	
	*M*	*SD*	*M*	*SD*
Age (years)	14.74	2.32	15.15	5.05
WASI FSIQ	87.5	16.27	122.27	7.78
SRS-SCI (*T*-score)	65.63	15.31	43.10	6.10
SRS-RRB (*T*-score)	64.56	12.80	47.70	6.88
Conners Inattention (*T*-score)	65.44	16.67	48.20	9.17
Conners Hyperactivity/Impulsivity (*T*-score)	55.88	14.56	46.50	10.14
Total intracranial volume (ml)	1480.53	191.51	1462.77	179.03
Head motion (mean relative framewise displacement)	0.15	0.07	0.15	0.05
Age of seizure onset (months)	13.47	12.98	–	–
E-Chess (current seizure severity score)	2.69	4.45	–	–
Tuber load (number)	21.71	15.26	–	–
				
Sex (% female)	63 %		83 %	–
Genetic mutation (% TSC2)	85 %		–	–
Current epilepsy medication (% medicated)	25 %		–	–

Note. WASI = Wechsler Adult Scale of Intelligence; FSIQ = Full Scale Intelligence Quotient; SRS = Social Responsiveness Scale; SCI = Social Communication Index; RRB = Restricted interests and repetitive behaviours; E-Chess = Early Childhood Epilepsy Severity Scale.

### 3.1 Behavioural group differences

TSC participants showed significantly lower FSIQ compared to controls (*β* = −36.66, *p* <.001), controlling for sex and age. TSC participants also showed significantly higher SRS-SCI, (*β* = 22.19, *p* <.001), SRS-RRB, (*β* = 16.43, *p* =.002), and Conners Inattention scores, (*β* = 16.81, *p* =.012), compared to controls. The two groups did not significantly differ in terms of Conners Hyperactivity/Impulsivity scores,(*β* = 8.50, *p* =.133). Within-group correlations between all behavioural variables can be found in Supplementary Table 1.

### 3.2 Neural group differences

Non-parametric permutations tests revealed significant fixel-wise differences between TSC and controls in all three FBA metrics (FDC, FD, and log-FC).

**Fig. 1:**
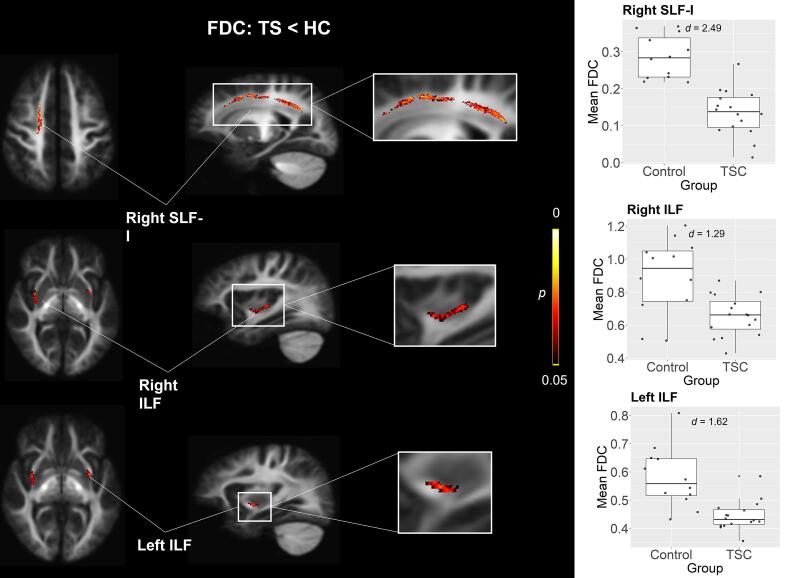
Comparison of FDC between TSC and Control participants. TSC participants show reduced FDC compared to Controls in right superior longitudinal fasciculus (SLF) and bilateral inferior longitudinal fasciculus (ILF).

TSC participants showed significantly reduced FDC in the dorsal branch of the right superior longitudinal fasciculus (SLF-I; *β* = −0.16, *p* =.033), left inferior longitudinal fasciculus (ILF; *β* = −0.24, *p* =.038), and right ILF (*β* = −0.15, *p* =.038) (see Fig. 1). Follow-up analyses in these three significant regions revealed that TSC showed both reduced FD (SLF-I: *β* = −0.13, *p* <.001; left ILF: *β* = −0.06, *p =*.012; right ILF: *β* = −0.08, *p* =.037) and reduced log-FC (SLF-I: *β* = −0.13, *p* <.004; left ILF: *β* = −0.15, *p <*.001; right ILF: *β* = −0.17, *p* =.004) in all three tracts compared to controls.

**Fig. 2:**
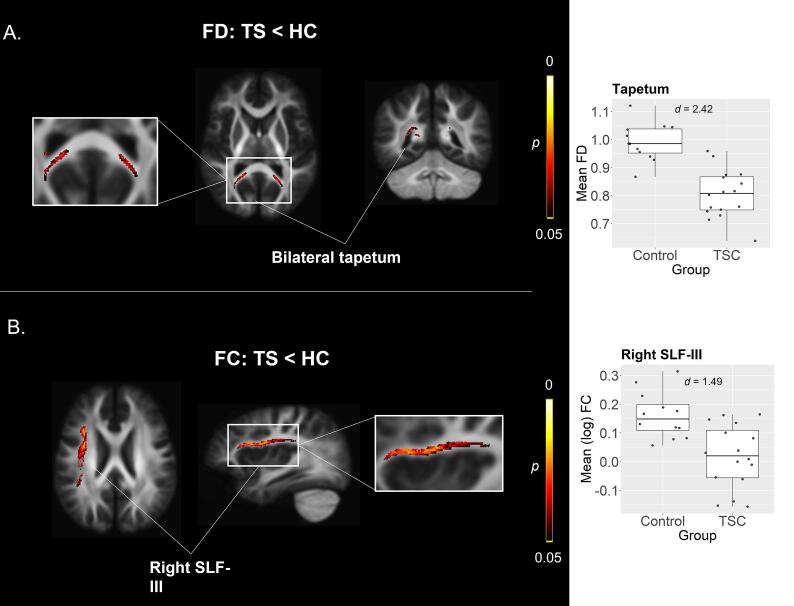
Comparison of FD and FDC between TSC and Control participants. (A) TSC participants show reduced FD in bilateral tapetum and (B) reduced FC in right SLF compared to Controls.

Whole-brain group comparison of FD showed that TSC participants also exhibited reduced FD in the tapetum bilaterally compared to controls (*β* = −0.17, *p* =.039) (Fig. 2A). Finally, TSC participants also showed significantly reduced log-FC in the ventral branch of the right superior longitudinal fasciculus (*β* = −0.16, *p* =.036; SLF-III) (Fig. 2B).

### 3.3 Brain-behaviour associations in TSC participants

Mean FDC, FD, and log-FC were extracted for each participant from the three significant regions showing reduced FDC in TSC (right SLF-I and bilateral ILF). Mean FD was extracted from bilateral tapetum and mean log-FC was extracted from right SLF-III.

Regression analyses (all controlling for age, sex, and scanner) within the TSC group revealed that FSIQ was not associated with FBA metrics in any of the identified regions, with all *p-*values > 0.0125.

**Fig. 3:**
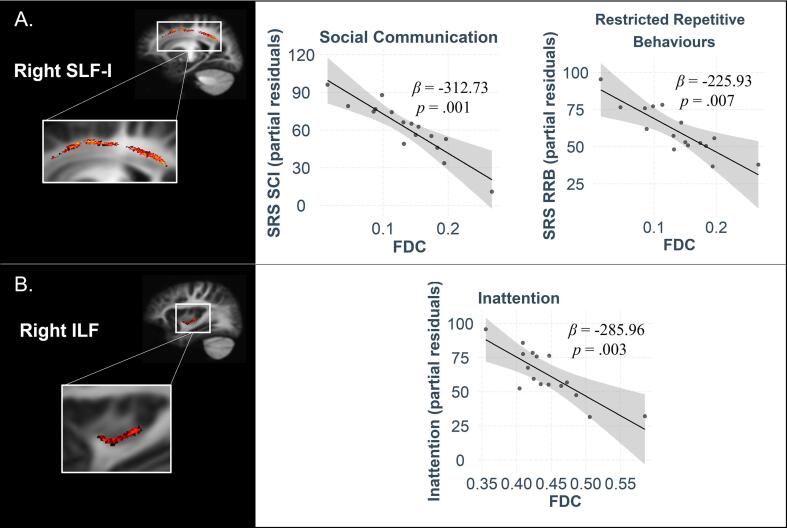
Brain-behaviour associations in TSC participants. (A) Association between FDC in right SLF and SRS-SCI as well as SRS-RRB and (B) association between FDC in right ILF and Conners Inattention score in TSC participants. Scatter plots show partial correlations controlling for age, sex, and scanner.

In contrast, SRS-SCI was significantly negatively associated with FDC in right SLF-I (*β* = −312.73, *p* =.001) (Fig. 3A). Further analyses revealed that this was due to a significant association between SRS-SCI and FD (*β* = 308.33, *p* <.001), but not FC (*β* = −24.39, *p* =.548) in this region. Moreover, SRS-RRB was significantly negatively associated with FDC in right SLF-I (*β* = −225.93, *p* =.007) (Fig. 3A). Similarly, this was driven by an association with FD (*β* = −223.03, *p* =.006), but not FC (*β* = −43.05, *p* =.183) in this region. Finally, there was a significant negative association between Conners Inattention score and FDC in the right ILF (*β* = −285.96, *p* =.003) (Fig. 3B); an effect once again underpinned by an association with FD (*β* = −263.77, *p* =.009), but not FC (*β* = −43.16, *p* =.583), in this region.

No behavioural measure was associated with FD in bilateral tapetum or with log-FC in SLF-III in the TSC group.

For completeness, we also tested brain-behaviour relationships in the control group; none of these were significant, with all *p*-values > 0.05.

Full regression outputs for all brain-behaviour analyses can be found in Supplementary Table S2.

### 3.4 Associations with clinical variables

FD in the bilateral tapetum was significantly associated with tuber load, *β* = −0.003, *p* =.037 (controlling for age, sex, and scanner), indicating that higher total number of tubers was related to reduced FD in this region (Supplementary Figure S1). Tuber load was not associated with any other FBA metrics in any of the remaining regions of interest, or with any behavioural variable of interest, all *p*s > 0.05. None of the FBA metrics in any regions were significantly associated with current epilepsy severity, as indexed by the E-Chess total score, *p* >.05. However, younger age of seizure onset was significantly associated with reduced FDC in right SLF-I (*β* = 0.001, *p* =.0180; Supplementary Figure S2), but not with any other FBA metrics.

## 4 Discussion

In this study we used fixel-based analysis to assess white matter changes in young individuals with a diagnosis of TSC compared to control participants without TSC, and tested whether these differences are related to neurodevelopmental symptoms on the autism and inattention spectrums. TSC participants showed fibre-specific reductions in FD, FC, and FDC within several white matter tracts compared to controls, and changes were selectively associated with behavioural symptoms in the TSC group. Specifically, reduced FDC in right SLF-I was associated with increased social communication difficulties and restricted, repetitive behaviours on the autism spectrum, while reduced FDC in left ILF was associated with higher symptoms of inattention. In contrast, white matter changes in affected regions were not associated with IQ, suggesting a specific role for FDC deficits in neurodevelopmental presentations in TSC. Finally, tuber load was associated with reduced FD in bilateral tapetum, while younger age at seizure onset was associated with reduced FDC in right SLF-I.

Our findings of altered white matter fibre properties complement previous work studying white matter changes in TSC using diffusion imaging. Numerous studies converge on the finding of altered diffusion characteristics measured specifically within tuber regions (Tsai et al., 2021, Jansen et al., 2003, Tiwari et al., 2012); however, there has been some inconsistency with respect to white matter regions appearing “normal” on conventional MR. While some studies have reported increased diffusivity (in terms of mean diffusivity or apparent diffusion coefficient) and decreased anisotropy, indicative of compromised structural integrity, in normal-appearing white matter in TSC compared to controls (Garaci et al., 2004, Makki et al., 2007), other studies have found no or only weak differences between groups in these extra-lesional regions (Karadag et al., 2005, Firat et al., 2006, Arulrajah et al., 2009). Further studies investigating a range of major white matter tracts throughout the brain have variably observed altered microstructure in several tracts such as corpus callosum (Peters et al., 2012, Krishnan et al., 2010), inferior (Ridler et al., 2001) and superior longitudinal (Taoka et al., 2020, Zikou et al., 2016) and uncinate (Prohl et al., 2019) fasciculi. The mixed findings within the TSC diffusion literature may reflect in part the shortcomings of conventional diffusion imaging techniques, which are typically unable to characterise microstructural properties in regions of multiple fibre orientations or differentiate between micro- and macrostructural properties of specific fibre bundles. Using a fixel-based approach in which we evaluated the whole connectome rather than an a priori selected subset of white matter regions, we were able to show that several white matter tracts are differentially affected with respect to fibre density (i.e., microstructure), fibre cross-section (i.e., macrostructure), or a combination of the two, in TSC.

TSC participants showed a significant reduction of FDC in SLF-I as well as bilateral ILF compared to controls. Importantly, FDC reductions in SLF-I were associated with autism symptoms, while FDC reductions in right ILF were associated with inattention in these patients, with both findings appearing to be primarily driven by changes in fibre density rather than cross-section. As the most dorsal branch of the SLF, SLF-I connects superior parietal with superior frontal regions and as such is an integral part of the dorsal frontoparietal attention network (De Schotten et al., 2011), which is particularly involved in spatial attention as well as higher aspects of motor behaviour (Parlatini et al., 2017). It is of particular interest that right SLF-I specifically related to autism symptoms, as white matter findings in idiopathic ASD in this tract are highly variable. Indeed, both increased (Cheung et al., 2009, Weinstein et al., 2011, Fitzgerald et al., 2018) and decreased (Fitzgerald et al., 2019, Poustka et al., 2012, Im et al., 2018, Barnea-Goraly et al., 2010, Kleinhans et al., 2012) structural connectivity within left and right SLF have been observed in ASD, although studies typically do not differentiate between the three branches of the SLF. Importantly however, several studies in idiopathic ASD have shown that reduced fractional anisotropy in right SLF was associated with autism spectrum social communication difficulties (Poustka et al., 2012), autism quotient (Bakhtiari et al., 2012), and RRB (Fitzgerald et al., 2019). These findings converge with our observations and suggest that microstructural integrity of SLF-I may be transdiagnostically involved in socio-communicative and behavioural difficulties on the autism spectrum. Moreover, reduced FDC in SLF-I was significantly associated with earlier age of seizure onset, which may be indicative of greater brain involvement in the individual TSC pathology, highlighting the role of early neurodevelopmental disruption in the emergence of autism symptoms in TSC (Bolton et al., 2002, Mitchell et al., 2021).

The ILF, in contrast, connects occipitotemporal regions with the anterior temporal lobe and is implicated in visuospatial processing as part of the sensory visual attention network. Our observation of an association between symptoms of inattention and reduced FDC in TSC largely aligns with findings in idiopathic ADHD. ADHD has been associated with white matter abnormalities in a range of tracts, including (but not limited to) bilateral ILF (Chen et al., 2016), and specific associations between persistent symptoms of inattention and ILF have been reported both in children (Svatkova et al., 2016) and adults (Versace et al., 2021, Konrad et al., 2012) (although it is worth noting that other tracts have also been implicated in inattentive symptoms in ADHD cohorts (Shaw et al., 2015; Wu et al., 2014). It has been suggested that altered connectivity via ILF may contribute to inattentive symptoms due to resultant perturbation of visually guided attentional processes (Versace et al., 2021), a notion supported by findings of ILF involvement in developmental learning difficulties that may be related to visual attention alterations (Chokron et al., 2021). Our findings suggest that the structural alterations in right ILF seen in TSC may underlie the severity of inattention in this population. Similarly to the observed association between SLF-I and autism symptoms, the link between ILF and inattention appears to be primarily driven by reduced fibre density (rather than cross-section). It is possible that this reflects a disruption of developmental processes in these regions resulting in either a reduced number of axons, or reduced diameter of axons, within the given fibres. The ensuing impairment in efficiently transmitting information along these tracts likely disrupts overall functioning within the relevant circuits, leading to a selective impact on separate behavioural domains. Of note, these white matter alterations were not related to general cognitive abilities, i.e., IQ, lending support to the notion that the downstream behavioural effects may be domain-specific.

We also observed changes in fibre density in bilateral tapetum and fibre cross-section in SLF-III in TSC participants which were unrelated to neurodevelopmental outcomes assessed in this study. White matter alterations in overlapping areas have been previously observed in TSC (Chen et al., 2016), although to our knowledge ours is the first study to disentangle effects of fibre density and cross-section on changes seen in these regions. The finding of reduced fibre cross-section in SLF-III is particularly striking, with the effect extending along most of this pathway. Importantly, SLF-III is known to be right-lateralised in typically developing individuals (De Schotten et al., 2011), although this observation is most pronounced in adulthood (Amemiya et al., 2021). Reduced cross-section of right SLF-III in TSC suggests that this lateralisation is not present in this population. This could have significant functional implications, as reduced or absent lateralization of white matter structures has previously been associated with specific developmental learning difficulties such as dyslexia (Banfi et al., 2019). We therefore cannot exclude the possibility that reduced fibre cross-section in right SLF-III observed in TSC participants is associated with behavioural difficulties not captured in this study.

Of note, our analyses of clinical variables revealed that the only white matter alteration related to tuber load was reduced fibre density in bilateral tapetum. In contrast, tuber load was not associated with any of the white matter changes related to neurodevelopmental symptoms, or indeed with behaviour itself. This may explain the previously reported poor predictive value of tuber load alone in predicting developmental outcomes in TSC (Jansen et al., 2008, Wong, 2006). It also highlights that TSC can result in specific white matter changes that may be independent of cortical tubers and lesions, but are nevertheless clinically meaningful. Furthermore, the fact that white matter properties in the identified regions were unrelated to behaviour in control participants suggests that the brain-behaviour relationships observed in TSC are not reflective of an extreme end of a continuum, but are rather the result of specific pathological processes underlying the emergence of developmental disorders in this condition. Nevertheless, it must be noted that we examined tuber load across the whole brain, and tract-specific information on tubers was not available to us. As our relatively small sample size precluded a more fine-grained analysis of regionally specific tuber locations in relation to white matter properties, it remains possible that the observed white matter alterations are related to tuber load in more specific and spatially relevant cortical regions.

This study is, to our knowledge, the first to use fixel-based analysis to characterise white matter alterations in TSC, and as such it provides novel insights into the biophysical properties of structurally disrupted white matter in this condition. Our findings suggest that the structural capacity to transfer information is compromised in selective white matter tracts – in particular right SLF, ILF, and bilateral tapetum – in TSC, and that autism and inattention symptoms are differentially related to fibre density cross-section in distinct regions. However, several limitations must be taken into account when considering these findings. First, the relatively small sample size limits the power of this study, such that more widespread abnormalities may not have been detected. Nevertheless, this may indicate that the regions highlighted in our study represent the most severely affected areas, which is further strengthened by the finding that these regions were independently related to symptom severity in the TSC sample alone. Furthermore, our sample had a wide age range spanning adolescence and early adulthood, which constitutes a sensitive time period during which white matter tracts are known to be undergoing continued development (Lebel and Beaulieu, 2011). While we controlled for potential age effects in our analyses, we were not able to explicitly interrogate age-dependent changes in the white matter abnormalities observed in the TSC sample. Future research in larger samples can usefully address this issue. In this context, longitudinal imaging will also be crucial in order to better understand progressive within-participant changes in structural connectivity and their relationship with neurodevelopmental symptoms in TSC throughout development. Our findings highlight potential mechanisms of interest that can shed light on the emergence of specific neurodevelopmental disorders and their neural substrates in TSC.

## CRediT authorship contribution statement

**Lucy D. Vanes:** Formal analysis, Methodology, Writing – original draft. **Charlotte Tye:** Conceptualization, Data curation, Funding acquisition, Methodology, Supervision, Writing – review & editing. **Jacques-Donald Tournier:** Formal analysis, Methodology, Writing – review & editing. **Anna J.E. Combes:** Data curation, Investigation, Writing – review & editing. **Elizabeth Shephard:** Data curation, Investigation, Writing – review & editing. **Holan Liang:** Data curation, Investigation, Writing – review & editing. **Gareth J. Barker:** Conceptualization, Investigation, Writing – review & editing. **Chiara Nosarti:** Conceptualization, Writing – review & editing. **Patrick Bolton:** Conceptualization, Funding acquisition, Investigation, Methodology, Supervision, Project administration, Writing – review & editing.

## Declaration of Competing Interest

The authors declare that they have no known competing financial interests or personal relationships that could have appeared to influence the work reported in this paper.
