# Supplementary material for: White matter disruptions related to inattention and autism spectrum symptoms in tuberous sclerosis complex
Source: Neuroimage Clin. 2022 Aug 25;36:103163. doi: 10.1016/j.nicl.2022.103163 (PMC9434133; doi:10.1016/j.nicl.2022.103163)
Supplement: Supplementary data 1 [file mmc1.pdf]

Supplementary Material

Tuberous Sclerosis 2000 Study Group

The members of the Tuberous Sclerosis 2000 Study Group are (affiliation at point of involvement): V Attard, A Clarke, FV Elmslie, AK Saggar, St George's Hospital, London; D Baines, BA Kerr, Royal Manchester Children's Hospital, Manchester; C Brayne, M Haslop, Y Granader, J Yates, University of Cambridge; I Carcani-Rathwell, C Connolly, M Clifford, H Liang, A Lydon, F McEwen, F Oluwo, H Rogers, C Srivastava, L Underwood, E Woodhouse, Institute of Psychiatry, Psychology & Neuroscience, King's College London; JA Cook, Sheffield Children's Hospital, Sheffield; C Falconer, St James's University Hospital, Leeds; DM Davies, JR Sampson, Institute of Medical Genetics, Cardiff; AE Fryer, Alder Hey Children's Hospital, Liverpool; PD Griffiths, University of Sheffield; N Higgins, Cambridge University Hospitals NHS Foundation Trust; A Hunt, Tuberous Sclerosis Association; WWK Lam, Western General Hospital, Edinburgh; JC Kingswood, Royal Sussex County Hospital, Brighton; ZH Miedzybrodzka, College of Life Sciences and Medicine, Aberdeen; H Crawford, PJ Morrison, Belfast City Hospital; FJK O'Callaghan, Great Ormond Street Hospital/Institute of Child Health, University College London; SG Philip, Birmingham Children's Hospital, Birmingham; S Seri, Aston Brain Centre, School of Life and Health Sciences, Aston University, Birmingham; F Sheerin, Oxford University Hospitals NHS Foundation Trust; R Sheehan-Dare, The General Infirmary, Leeds; CH Shepherd, Craigavon Area Hospital, Craigavon.

Assessment of tuber load

Tuber load was ascertained by three expert neuroradiologists who reviewed and elected the best available clinical MRI scan (usually the most recent). The scans were reviewed and rated without knowledge of other clinical details using a pre-specified coding system. Number and location of tubers, as well as sup-endymal nodules and sub-ependymal giant cell astrocytomas, was ascertained by visual inspection for each of the following gyral locations, for each hemisphere separately, based on a detailed cross-sectional anatomical atlas (Duvernoy 1999):

Superior frontal gyrus; middle frontal gyrus; inferior frontal gyrus; precentral gyrus; orbitofrontal cortex; dorsolateral prefrontal cortex; insula; superior temporal gyrus; middle temporal gyrus; inferior temporal gyrus; fusiform gyrus; parahippocampal gyrus; superior parietal gyrus; inferior parietal gyrus; postcentral gyrus; superior occipital gyrus; middle or lateral occipital gyrus; inferior occipital gyrus; cerebellar hemispheres; vermis.

Sensitivity analyses

For TSC participants, a binary medication status variable indicated whether or not participants were currently prescribed antiepileptic medication.

Total intracranial volume (TIV) was derived from anatomical T1-weighted scans acquired in the same session (acquisition parameters: TR = 7321 ms, TE = 3 ms, TI = 400 ms, field of view = 270, slice thickness = 1.2 mm, 196 slices) using the Computational Anatomy Toolbox (CAT12) (Gaser, Dahnke et al. 2022).

In-scanner head motion was quantified as mean relative framewise displacement across all acquired diffusion images, as derived from the motion correction procedure using FSL's *eddy_cuda* tool as implemented in *fsldwipreproc*.

Sensitivity analyses were run on all extracted regions showing significant group differences in FDC, FD, and log-FC, whereby TIV, head motion, or medication status were included separately as additional covariates (next to age, sex, scanner) of non-interest. Since relevant medication status was only available for TSC participants, sensitivity analyses including medication status as additional covariate were only run for brain-behaviour associations (within the TSC group). Instead, group differences in behavioural and fixel-based metrics were tested after excluding the 4 participants currently prescribed medication (and controlling for age, sex, and scanner).

All observed behavioural group differences remained significant after excluding medicated participants (FSIQ: β = -37.21, p < .001; SRS-SCI: β = 25.16, p < .001; SRS-RRB: β = 18.17, p = 003; Conners Inattention: β = 15.60, p = .015).

All observed fixel-based group differences remained significant after excluding medicated participants (FDC in right SLF-I: β = -0.16, p < .001; FDC in right ILF: β = -0.21, p = .003; FDC in left ILF: β = -0.14, p < .001; FD in bilateral tapetum: β = -0.17, p < .001; log-FC in right SLF-III: β = -0.17, p < .001), and after controlling for TIV (FDC in right SLF-I: β = -0.16, p < .001; FDC in right ILF: β = -0.21, p = .004; FDC in left ILF: β = -0.13, p < .001; FD in bilateral tapetum: β = -0.18, p < .001; log-FC in right SLF-III: β = -0.16, p < .001) and after controlling for head motion (FDC in right SLF-I: β = -0.15, p < .001; FDC in right ILF: β = -0.23, p = .004; FDC in left ILF: β = -0.14, p < .001; FD in bilateral tapetum: β = -0.17, p < .001; log-FC in right SLF-III: β = -0.16, p < .001).

All within-TSC brain-behaviour associations remained significant after controlling for TIV, head motion, or medication status (see Table S2).

References

Duvernoy, H. M. (1999). The human brain: surface, three-dimensional sectional anatomy with MRI, and blood supply, Springer Science & Business Media.

Gaser, C., R. Dahnke, P. M. Thompson, F. Kurth and E. Luders (2022). "CAT-a computational anatomy toolbox for the analysis of structural MRI data." bioRxiv.

Table S1. Within-group correlations between behavioural variables

	WASI FSIQ	SRS-SCI	SRS-RRB	Conners Inattention
**Group: TS**				
WASI FSIQ				
SRS-SCI	-0.48			
SRS-RRB	-0.42	**0.82*** **		
Conners Inattention	-0.12	0.32	**0.55***	
Conners Hyperactivity/Impulsivity	0.29	0.19	0.48	**0.56***
**Group: Control**				
WASI FSIQ				
SRS-SCI	<0.001			
SRS-RRB	0.05	**0.87*****		
Conners Inattention	0.04	**0.84****	**0.85****	
Conners Hyperactivity/Impulsivity	-0.02	0.60	**0.69***	**0.76***

*. Correlation is significant at 0.05 level.
**. Correlation is significant at 0.01 level.
***. Correlation is significant at 0.001 level.
Note. WASI = Wechsler Adult Scale of Intelligence; FSIQ = Full Scale Intelligence Quotient; SRS = Social Responsiveness Scale; SCI = Social Communication Index; RRB = Restricted interests and repetitive behaviours.

Table S2. Regression output for fixel-based predictors of interest for main and sensitivity analyses of brain-behaviour associations in TSC participants.

Predictor of interest	Main analysis			Sensitivity analyses								
	Main covariates: age, sex, scanner			Main covariates + TIV			Main covariates + motion			Main covariates + medication status		
	β	*SE*	*p*	β	*SE*	*p*	β	*SE*	*p*	β	*SE*	*p*
Dependent variable: **FSIQ**												
FDC right SLF-I	124.3	117.6	.313	124.52	116.5	.310	120.53	128.0	.369	120.14	135.5	.396
FDC right ILF	65.40	36.02	.097	57.67	41.15	.191	64.82	37.62	.116	78.00	37.14	.062
FDC left ILF	121.43	94.44	.225	118.00	93.82	.237	139.00	115.3	.256	127.59	97.61	.220
FD tapetum	35.99	56.03	.534	114.23	61.85	.095	33.69	59.01	.581	35.27	58.18	.558
Log-FC left ILF	45.31	54.18	.421	36.24	55.37	.528	48.18	56.56	.414	46.55	56.13	.426
Dependent variable: **SRS-SCI**												
FDC right SLF-I	-312.7	70.48	.001	-312.8	72.85	.002	-346.3	65.92	<.001	-263.8	72.12	.004
FDC right ILF	-8.79	39.07	.826	-4.08	44.95	.929	-9.29	40.88	.825	-34.00	33.23	.331
FDC left ILF	-67.87	94.41	.487	-66.86	98.62	.513	-110.8	112.8	.349	-87.36	78.01	.289
FD tapetum	4.34	54.41	.938	-10.55	71.47	.886	2.45	57.43	.967	7.18	46.36	.880
Log-FC left ILF	42.74	51.70	.426	47.31	54.47	.405	44.81	54.22	.428	38.42	43.81	.401
Dependent variable: **SRS-RRB**												
FDC right SLF-I	-225.9	69.03	.007	-226.0	71.67	.010	-245.9	71.47	.006	-220.2	79.43	.019
FDC right ILF	-31.76	30.81	.325	-33.08	35.54	.374	-31.95	32.32	.346	-46.02	30.13	.158
FDC left ILF	-94.02	74.36	.232	-93.23	77.69	.258	-132.9	88.05	.162	-103.6	72.19	.181
FD tapetum	-14.61	44.62	.750	-37.44	57.75	.531	-15.36	47.20	.752	-13.31	44.26	.770
Log-FC left ILF	53.72	40.80	.215	58.05	42.76	.204	54.74	42.95	.231	51.78	40.36	.228
Dependent variable: **Conners Inattention**												
FDC right SLF-I	-264.5	114.0	.041	-264.4	116.6	.047	-241.6	121.3	.074	-356.3	111.2	.009
FDC right ILF	-66.64	41.70	.138	-94.75	43.14	.053	-64.46	41.19	.149	-65.05	45.73	.185
FDC left ILF	-286.0	4.84	.003	-288.6	74.50	.003	-303.7	91.13	.008	-282.7	78.22	.005
FD tapetum	-59.67	61.76	.355	-57.29	81.59	.499	-51.21	62.33	.430	-60.63	63.81	.364
Log-FC left ILF	64.99	59.85	.301	60.18	63.18	.363	58.27	60.08	.356	66.54	61.78	.307

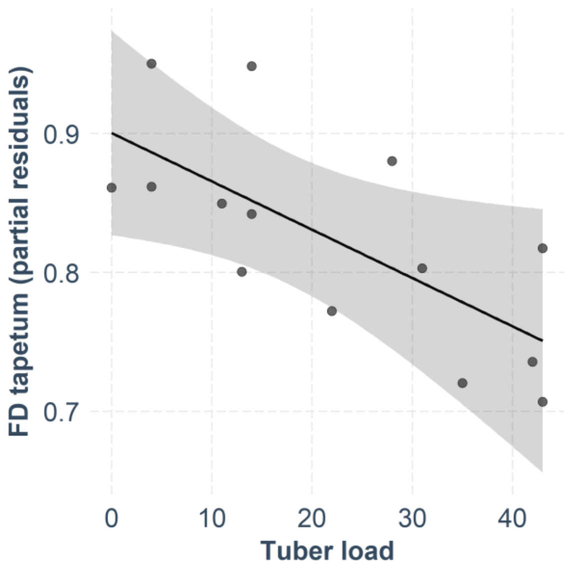

Figure S1. Association between fibre density (FD) in bilateral tapetum and tuber load, controlling for age, sex, and scanner.

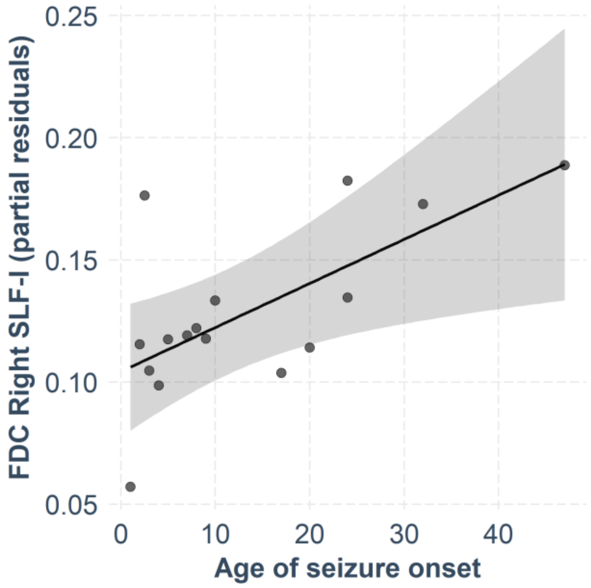

Figure S2. Association between fibre density cross-section (FDC) in right dorsal superior longitudinal fasciculus (SLF-I) and age at seizure onset, controlling for age, sex, and scanner.
